# Galactosyl carbohydrate residues on hematopoietic stem/progenitor cells are essential for homing and engraftment to the bone marrow

**DOI:** 10.1038/s41598-019-43551-6

**Published:** 2019-05-09

**Authors:** Soichiro Takagaki, Rieko Yamashita, Noriyoshi Hashimoto, Kazushi Sugihara, Kanako Kanari, Keisuke Tabata, Toshikazu Nishie, Shogo Oka, Masanori Miyanishi, Chie Naruse, Masahide Asano

**Affiliations:** 1 Division of Transgenic Animal Science, Advanced Science Research Center, Kanazawa University, Kanazawa, 920-8640 Japan; 2 Institute of Laboratory Animals, Graduate School of Medicine, Kyoto University, Kyoto, 606-8501 Japan; 3 Department of Biological Chemistry, Human Health Sciences, Graduate School of Medicine, Kyoto University, Kyoto, 606-8507 Japan; 4 Laboratory for Organismal Patterning, RIKEN Center for Biosystems Dynamics Research, Kobe, 650-0047 Japan

**Keywords:** Glycobiology, Haematopoietic stem cells

## Abstract

The role of carbohydrate chains in leukocyte migration to inflamed sites during inflammation and trafficking to the lymph nodes under physiological conditions has been extensively characterized. Here, we report that carbohydrate chains also mediate the homing and engraftment of hematopoietic stem/progenitor cells (HSPCs) to the bone marrow (BM). In particular, we found that transplanted BM cells deficient in β-1,4-galactosyltransferase-1 (β4GalT-1) could not support survival in mice exposed to a lethal dose of irradiation. BM cells obtained from mice deficient in β4GalT-1 showed normal colony-forming activity and hematopoietic stem cell numbers. However, colony-forming cells were markedly rare in the BM of recipient mice 24 h after transplantation of β4GalT-1-deficient BM cells, suggesting that β4GalT-1 deficiency severely impairs homing. Similarly, BM cells with a point mutation in the UDP-N-acetylglucosamine 2-epimerase/N-acetylmannosamine kinase gene, encoding a key enzyme in sialic acid biosynthesis, showed mildly impaired homing and engraftment abilities. These results imply that the galactosyl, but not sialyl residues in glycoproteins, are essential for the homing and engraftment of HSPCs to the BM. These findings suggest the possibility of modifying carbohydrate structures on the surface of HSPCs to improve their homing and engraftment to the BM in clinical application.

## Introduction

The mechanisms and processes underlying leukocyte migration to inflamed sites during inflammation and trafficking to the lymph nodes under physiological conditions have been well characterized. For example, selectins and carbohydrate ligands such as sialyl Lewis x (sLe^x^) and 6-sulfo sLe^x^ are now known to facilitate the leukocyte–endothelium interaction, the first step in these processes^1^. Accordingly, mice deficient in both E- and P-selectins, which bind to sLe^x^ ^2^, show severe impairment in leukocyte migration to inflamed sites^3,4^, while mice deficient in L-selectin, which binds to 6-sulfo sLe^x^ ^5^, show compromised leukocyte trafficking to the lymph nodes^6^. In addition, insight into the roles of the selectin ligand biosynthesis pathway in leukocyte trafficking has also been obtained using mouse models. For example, mice deficient in both fucosyl transferase-IV and VII, which are responsible for the synthesis of sLe^x^ and 6-sulfo sLe^x^, show impaired selectin-dependent leukocyte recruitment and lymphocyte homing^7^. Similarly, we have shown that mice deficient in β-1,4-galactosyltransferase-1 (β4GalT-1) exhibit reduced inflammatory responses^8^ and delayed wound healing^9^ due to impaired leukocyte infiltration following repressed biosynthesis of endothelial selectin ligands. These results indicate that β4GalT-1 is also involved in the biosynthesis of selectin ligands. However, β4GalT-1 deficiency did not affect leukocyte trafficking to the lymph nodes^8^.

The role of carbohydrate chains in the homing and engraftment of hematopoietic stem/progenitor cells (HSPCs) to the bone marrow (BM) remains largely undefined, although previous studies have suggested the role of galactosyl and mannosyl residues^10^. Notably, several cell adhesion molecules, including integrin family members, are known to be essential in homing and engraftment^11–13^. For example, very late antigen-4 (VLA-4, α4β1 integrin) and VLA-5 (α5β1 integrin) in HSPCs bind to vascular cell adhesion molecule-1 (VCAM-1) and fibronectin in the BM, respectively^14,15^. Accordingly, antibodies against these molecules^16,17^ or genetic deletion of hematopoietic β1 integrin^18^ severely impair HSPC homing and engraftment. Leukocyte function-associated antigen-1 (LFA-1, αLβ2 integrin) and lymphocyte Peyer’s patch cell adhesion molecule-1 (LPAM-1, α4β7 integrin) in HSPCs interact with intercellular adhesion molecule-1 (ICAM-1) and mucosal addressin cell adhesion molecule-1 (MadCAM-1) in the BM, respectively, which have also been shown to promote homing and engraftment^17,19^. Several other factors are also known to be important in HSPC homing/engraftment, including hyaluronic acid/CD44^20^, guanosine triphosphatases rac1 and rac2^21^, osteopontin^22^, sphingosine 1-phosphate receptor^23^, prostaglandin E2^24^, and membrane-bound SCF/c-Kit^25^.

Moreover, stromal-derived factor 1 (SDF-1, also known as CXCL12) acts as a major HSPC chemoattractant through its receptor CXCR4^26^, and mediates HSPC homing and engraftment in cooperation with LFA-1, VLA-4, and VLA-5^17^; accordingly, deficiency in SDF-1 or CXCR4 severely compromises these events^27,28^. Despite this extensive research and identification of the key players involved, the role of carbohydrate chains in the function of these cell adhesion and chemoattractant molecules is unknown. However, there is ample evidence to indicate the involvement of endothelial selectins and their carbohydrate ligands. For example, lethally irradiated mice deficient in both P- and E-selectin do not survive when transplanted with 5 × 10^4^ wild type BM cells, the minimum number that would otherwise be required, whereas approximately 80% of wild-type recipient mice do. Homing in P/E-selectin double-knockout recipient mice is mildly reduced, and further compromised by treatment with antibodies to VCAM-1^29^. E-selectin ligands and α4 integrin expressed in HSPCs have also been shown to cooperate in HSPC homing and engraftment^30^. Overall, these results suggest that VLA-4/VCAM-1 and CXCR4/SDF-1 are dominant players in HSPC homing/engraftment, with LFA-1/ICAM-1 and selectin ligands/endothelial selectins playing more minor roles.

The aim of the present study was to elucidate the role of carbohydrate chains in HSPC homing/engraftment to the BM using carbohydrate-modified mice. Specifically, we transplanted HSPCs deficient in β4GalT-1 (*β4GalT-1*^−/−^) in lethally irradiated mice, and determined the effect on survival of the mice, and tracked their homing ability to the BM compared to those of BM cells harboring a point mutation in UDP-N-acetylglucosamine 2-epimerase/N-acetylmannosamine kinase (GNE), a key enzyme involved in sialic acid biosynthesis. In addition, homing and engraftment of immature fetal liver HSPCs of *β4GalT-1*^−/−^ mice to adult BM were examined. Overall, these results provide insights into the relative roles of galactosyl and sialyl residues compared to the more well-known roles of selectin ligands in HSPC homing and engraftment to the BM.

## Results

### HSPC population and colony-forming activities of BM cells

**Figure 1:**
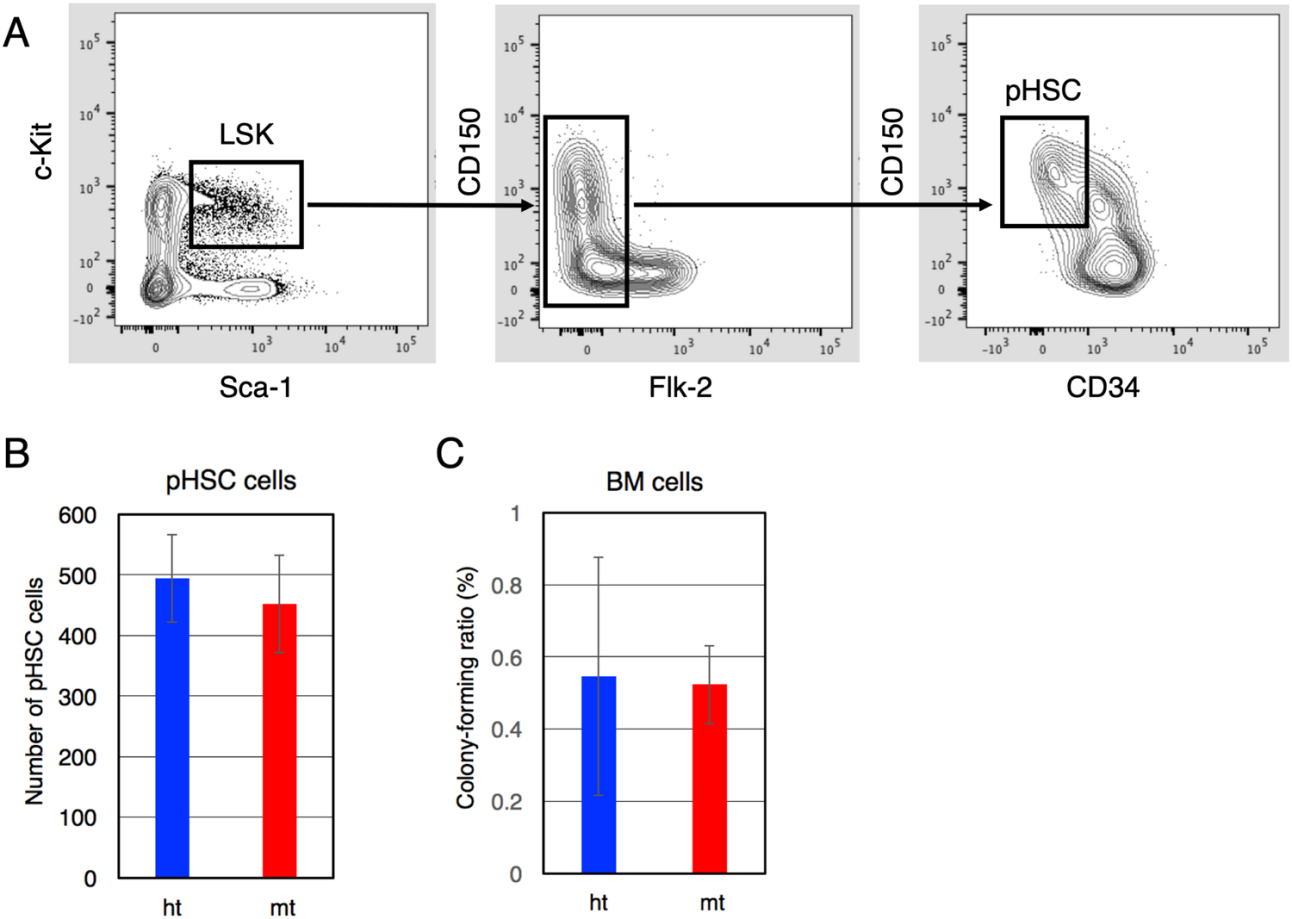
Hematopoietic stem cell (HSC) population and colony-forming activity of bone marrow (BM) cells. (**A**) Flow cytometry analysis of BM cells using various antibodies against indicated cell surface markers. pHSCs were defined as lineage^−^, Sca-1^+^, c-Kit^+^, Flk-2^−^, CD150^+^, CD34^−/low^. (**B**) The number of HSCs examined by flow cytometry (A) per 1 × 10^6^ BM cells obtained from *β4GalT-1*^+/−^ (ht, n = 4) and *β4GalT-1*^−/−^ mice (mt, n = 4). (**C**) Colony-forming ratios of BM cells obtained from *β4GalT-1*^+/−^ (ht, n = 9) and *β4GalT-1*^−/−^ mice (mt, n = 9). Error bars indicate the S.D.

Hematopoietic cells, including lymphocytes, neutrophils, monocytes, and red blood cells, are present in the peripheral blood of *β4GalT-1*^−/−^ mice, although leukocytosis and mild anemia are observed^8^. The T/B cell ratio and CD4/CD8 cell ratio were normal in the spleen and thymus of these mice, respectively (data not shown). To examine the hematopoietic stem cell (HSC) population in BM cells, we performed flow cytometry analysis using various cell surface markers (Fig. 1A). The number of pHSCs^31^ defined as lineage (Ter-119, B220, CD3e, CD4, CD8a, Gr-1, and CD11b, IL-7R)-negative, Sca-1^+^, c-Kit^+^, Flk-2^−^, CD150^+^, CD34^−/low^ was comparable between *β4GalT-1*^−/−^and *β4GalT-1*^+/−^ mice (Fig. 1B). In addition, colony formation by BM cells from *β4GalT-1*^−/−^ mice was comparable to that of BM cells from *β4GalT-1*^+/−^ mice (Fig. 1C), consistent with our previous reports showing that colony formation in the presence of granulocyte-colony stimulating factor or IL-3 was comparable between these mice^8^. These results imply that the HSC population and colony-forming activity of HSPCs are not affected by β4GalT-1 deficiency.

**Figure 2:**
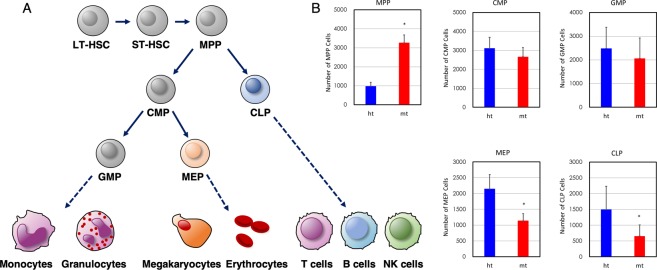
Hematopoietic stem/progenitor cell (HSPC) differentiation. (**A**) Illustration of HSPC differentiation to terminal blood cells^32^. LT-HSC; long term-HSC, ST-HSC; short term-HSC, MPP; multipotent progenitor, CLP; common lymphoid progenitor, CMP; common myeloid progenitor, GMP; granulocyte-macrophage progenitor, MEP; megakaryocyte-erythroid progenitor. (**B**) The numbers of MPP, CMP, CLP, GMP and MEP examined by flow cytometry per 1 × 10^6^ BM cells obtained from *β4GalT-1*^+/−^ (ht, n = 6) and *β4GalT-1*^−/−^ mice (mt, n = 6). Error bars indicate S.D. *p < 0.05. MPP; Lin^−^, IL-7R^−^, c-kit^+^, Sca-1^+^, CD34^+^, Flk-2^−^, CMP; Lin^−^, IL-7R^−^, c-kit^+^, Sca-1^−^, CD34^+^, FcgR^low^, CLP; Lin^−^, IL-7R^+^, Flk-2^+^, GMP; Lin^−^, IL-7R^−^, c-kit^+^, Sca-1^−^, CD34^+^, FcgR^+^, MEP; Lin^−^, IL-7R^−^, c-kit^+^, Sca-1^−^, CD34^−^, FcgR^low^, as described in the gating scheme (Fig. S1).

To further examine HSPC populations in BM cells, as illustrated in Fig. 2A ^32^, we performed flow cytometry analysis according to the gating scheme (Fig. S1), as described previously^31^. The numbers of multipotent progenitor (MPP), common myeloid progenitor (CMP), common lymphoid progenitor (CLP), granulocyte-macrophage progenitor (GMP), and megakaryocyte-erythroid progenitor (MEP) are shown in Fig. 2B. The number of MPP increased 3-fold in *β4GalT-1*^−/−^ BM cells compared to *β4GalT-1*^+/−^ BM cells. Although the numbers of CMP and GMP were equivalent between the genotypes, the numbers of MEP and CLP decreased by approximately half in *β4GalT-1*^−/−^ BM cells compared to *β4GalT-1*^+/−^ BM cells. These results suggest that HSC differentiation was disturbed to some extent downstream of MPP.

### Impaired hematopoietic reconstitution by *β4GalT-1*^−/−^ BM cells

We previously reported that β4GalT-1 is involved in the biosynthesis of selectin ligands, indicated by the impairment of inflammatory responses and wound healing in *β4GalT-1*^−/−^ mice^8,9^. As the carbohydrate chains in HSPCs may also regulate homing and engraftment to the BM, we first examined the extent of hematopoietic reconstitution in lethally irradiated mice (Table 1), which generally die within 10 days unless transplanted with functional BM cells. Remarkably, the lethally irradiated mice intravenously transplanted with *β4GalT-1*^−/−^ BM cells also died within 10 days, whereas those transplanted with *β4GalT-1*^+/−^ cells survived for more than 60 days. Conversely, lethally irradiated *β4GalT-1*^−/−^ mice transplanted with BM cells from wild type C57BL/6 mice also survived for more than 60 days, while lethally irradiated *β4GalT-1*^−/−^ mice transplanted with *β4GalT-1*^−/−^ BM cells died within 10 days. Collectively, these results indicated that β4GalT-1 activity in transplanted BM cells, but not in recipient mice, is critical for hematopoietic reconstitution. It is known that BM cells engraft to the BM more efficiently when they are directly transplanted into the BM cavity using the intra-BM transplantation (IBM-BMT) method^33^. Similar to the intravenous BM transplantation (IV-BMT), lethally irradiated wild type mice transplanted with BM cells from *β4GalT-1*^−/−^ mice by IBM-BMT died within 10 days, indicating that *β4GalT-1*^−/−^ BM cells could not reconstitute hematopoiesis even when the IBM-BMT method was used.

**Table 1:** Survival ratio of recipient mice after IV-BMT and IBM-BMT of β4GalT-1-deficient BMCs.

BMT methods	Donor BMCs	Recipient mice	Survival ratio (alive/total)				
			0*	5*	10*	15*	60*
IV-BMT	β4GalT-1 −/−	C57BL/6 (wt)	3/3	3/3	0/3	0/3	0/3
	β4GalT-1 +/−	C57BL/6 (wt)	4/4	4/4	4/4	4/4	4/4
	β4GalT-1 −/−	β4GalT-1 −/−	2/2	2/2	0/2	0/2	0/2
	C57BL/6 (wt)	β4GalT-1 −/−	3/3	3/3	3/3	3/3	3/3
	no	C57BL/6 (wt)	3/3	3/3	0/3	0/3	0/3
IBM-BMT	β4GalT-1 −/−	C57BL/6 (wt)	4/4	4/4	0/4	0/4	0/4
	β4GalT-1 +/−	C57BL/6 (wt)	5/5	5/5	5/5	5/5	5/5
	no	C57BL/6 (wt)	4/4	3/4	0/4	0/4	0/4
IV-BMT	β4GalT-1 −/−	NOD/SCID	4/4	4/4	0/4	0/4	
	β4GalT-1 +/−	NOD/SCID	4/4	4/4	4/4	4/4	
	no	NOD/SCID	2/2	1/2	0/2	0/2	

^*^Days after BMT.

We then transplanted a mixture of *β4GalT-1*^−/−^ and *β4GalT-1*^+/−^ BM cells to ensure survival (Table 2), and the former cells were labeled with green fluorescent protein (GFP) to enable tracking. Strikingly, GFP-positive cells were rarely detected in the spleen, thymus, and peripheral blood of recipient mice 9 weeks after transplantation even when 90% of the transplanted cells were *β4GalT-1*^−/−^ (Table 2, Exp. 2), whereas more than 90% of the cells in these tissues were GFP-positive when only GFP-positive wild type cells were transplanted (Table 2, Exp. 4). These results suggest that *β4GalT-1*^−/−^ BM cells are impaired in homing and engraftment after transplantation.

**Table 2:** Engraftment of mixed donor bone marrow cells (BMCs).

Tissues	Ratio of GFP-positive cells (%)			
	Exp 1	Exp 2	Exp 3	Exp 4
Peripheral blood	0.00	0.00	0.00	92.90
Spleen	0.04	0.46	0.00	95.05
Thymus	0.01	0.00	0.00	98.60

Exp 1; 1.0 × 10^7^ mt/GFP BMCs + 1.0 × 10^7^ ht BMCs.

Exp 2; 1.8 × 10^7^ mt/GFP BMCs + 0.2 × 10^7^ ht BMCs.

Exp 3; 0.4 × 10^7^ ht BMCs.

Exp 4; 2.0 × 10^7^ wt/GFP BMCs.

9 weeks after transplantation.

### Colony formation by transplanted *β4GalT-1*^−/−^ BM cells

**Figure 3:**
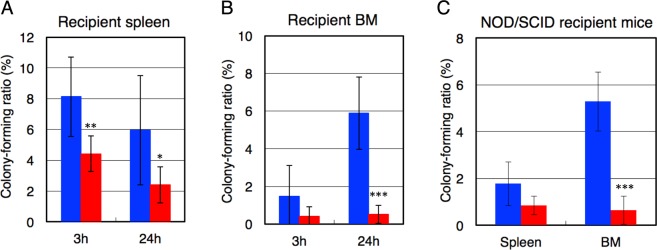
Homing of transplanted *β4GalT-1*^−/−^ BM cells. (**A**,**B**) Colony-forming ratios of splenocytes (**A**) and BM cells (**B**) obtained from wild-type recipient mice transplanted with *β4GalT-1*^+/−^ (n = 6–8) and *β4GalT-1*^−/−^ BM cells (n = 6–8), as measured 3 h and 24 h after transplantation. (**C**) Colony-forming ratios of splenocytes and BM cells obtained from NOD/SCID recipient mice transplanted with *β4GalT-1*^+/−^ (n = 4) and *β4GalT-1*^−/−^ BM cells (n = 4), as measured 24 h after transplantation. Blue bars, *β4GalT-1*^+/−^ cells; red bars, *β4GalT-1*^−/−^ cells. Error bars indicate the S.D. *p < 0.05, **p < 0.01, and ***p < 0.001.

To examine the homing ability of the transplanted HSPCs, splenocytes and BM cells were collected from the recipient mice at 3 h and 24 h after IV-BMT, and analyzed using a colony formation assay. Colony-forming transplanted *β4GalT-1*^−/−^ cells were approximately 0.5- and 0.3-fold as abundant in recipient splenocytes as in the transplanted *β4GalT-1*^+/−^ cells at 3 h and 24 h after IV-BMT, respectively. Similarly, the colony-forming ratio of transplanted *β4GalT-1*^+/−^ cells increased from 1.5% to 6% between 3 h and 24 h, but that of transplanted *β4GalT-1*^−/−^ cells was less than 0.5% (Fig. 3A,B). These results indicated that β4GalT-1 deficiency severely impaired the homing ability of transplanted HSPCs to the BM.

In these experiments, C57BL/6 mice were used as the recipient, although the donor cells were harvested from *β4GalT-1*^−/−^ or *β4GalT-1*^+/−^ mice on a mixed 129/Sv and C57BL/6 genetic background^34^. Nevertheless, the transplantation must be immunologically compatible, since both 129/Sv and C57BL/6 mice have an H-2^b^ haplotype. To completely exclude potential immunological effects, we also used immunodeficient NOD/SCID mice as the recipient for comparison. Lethally irradiated NOD/SCID mice transplanted with *β4GalT-1*^−/−^ BM cells also died within 10 days (Table 1), and the number of colony-forming transplanted cells in recipient BM cells was severely reduced compared with that of transplanted *β4GalT-1*^+/−^ cells (Fig. 3C). These results indicate that the homing deficiency of *β4GalT-1*^−/−^ BM cells was not due to immunological rejection.

**Figure 4:**
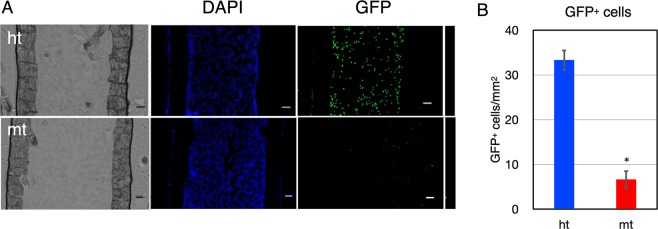
*In situ* observation of transplanted donor-derived bone marrow (BM) cells. (**A**) Frozen sections of the femur of wild-type recipient mice 24 h after BMT of GFP-labeled* β4GalT-1*^+/−^ (upper, ht) and* β4GalT-1*^−/−^ (lower, mt) BM cells. Bright field (left), DAPI (middle), GFP (right). Scale bar, 100 μm. (**B**) The number of GFP-positive cells per mm^2^ in (**A**) from *β4GalT-1*^+/−^ (ht, n = 3) and *β4GalT-1*^−/−^ (mt, n = 3) mice. *p < 0.05.

Lethally irradiated wild-type recipient mice were transplanted with GFP-labeled BM cells from *β4GalT-1*^−/−^ and *β4GalT-1*^+/−^mice and the femur of each recipient mouse was prepared 24 h after BMT. We observed GFP-labeled BM cells in the recipient femur using fluorescence microscopy (Fig. 4A). Abundant GFP-positive *β4GalT-1*^+/−^cells were observed in the BM of recipient mice, whereas *β4GalT-1*^−/−^ cells were rarely observed. Quantitative analysis showed that approximately 0.2-fold *β4GalT-1*^−/−^ cells adhered to the BM of recipient mice compared with *β4GalT-1*^+/−^ cells (Fig. 4B). These observations also support the notion that the homing ability of *β4GalT-1*^−/−^ HSPCs was severely impaired.

### Impaired hematopoietic reconstitution by *β4GalT-1*^−/−^ fetal liver cells

**Figure 5:**
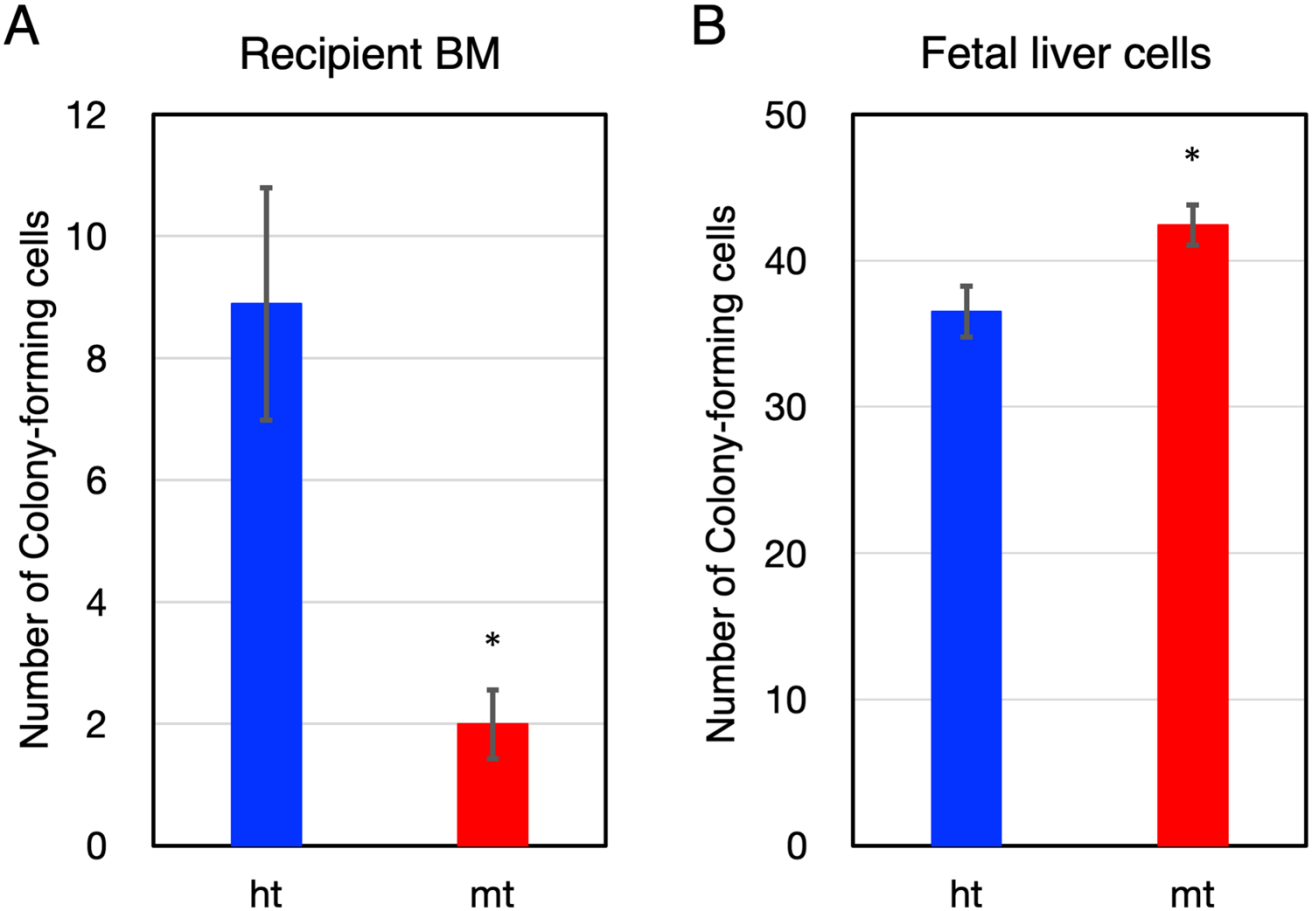
Homing of transplanted *β4GalT-1*^−/−^ fetal liver cells. (**A**) The number of colony-forming cells of BM cells obtained from wild-type recipient mice transplanted with *β4GalT-1*^+/−^ (ht, n = 5) and *β4GalT-1*^−/−^ (mt, n = 6) fetal liver cells, as measured 24 h after transplantation. (**B**) The number of colony-forming cells of fetal livers obtained from *β4GalT-1*^+/−^ (ht, n = 6) and *β4GalT-1*^−/−^ (mt, n = 8) E14.5 embryos. *p < 0.05.

During embryonic development, hematopoiesis mainly proceeds in the fetal liver^35,36^. We also examined homing and engraftment of immature HSPCs in the fetal liver of embryonic stage 14.5 (E14.5). Lethally irradiated wild-type mice transplanted with *β4GalT-1*^−/−^ fetal liver cells also died within 10 days, whereas those transplanted with *β4GalT-1*^+/−^ cells survived for more than 15 days (Table 3). Furthermore, the colony-forming ratio of transplanted *β4GalT-1*^−/−^ fetal liver cells were approximately 0.22-fold as abundant in recipient BM as they were in transplanted *β4GalT-1*^+/−^ fetal liver cells (Fig. 5A). On the other hand, direct colony-forming activity of fetal liver cells was slightly (1.2-fold), but significantly higher in *β4GalT-1*^−/−^ mice than it was in *β4GalT-1*^+/−^mice (Fig. 5B). As the difference in colony-forming activity of the fetal liver among the genotypes was small, it may not have affected the homing and engraftment activity. Therefore, homing and engraftment of immature HSPCs in the fetal liver of *β4GalT-1*^−/−^mice was also impaired similar to their mature HSPCs in the BM.

**Table 3:** Survival ratio of recipient mice after transfer of β4GalT-1-deficient fetal liver cells.

Donor fetal liver cells	Recipient mice	Survival ratio (alive/total)			
		0*	5*	10*	15*
β4GalT-1 −/−	C57BL/6 (wt)	5/5	5/5	0/5	0/5
β4GalT-1 +/−	C57BL/6 (wt)	7/7	7/7	6/7	6/7
no	C57BL/6 (wt)	2/2	2/2	0/2	0/2

^*^Days after transplantation of fetal liver cells.

### Lectin blot of lineage-negative *β4GalT-1*^−/−^ BM cells

**Figure 6:**
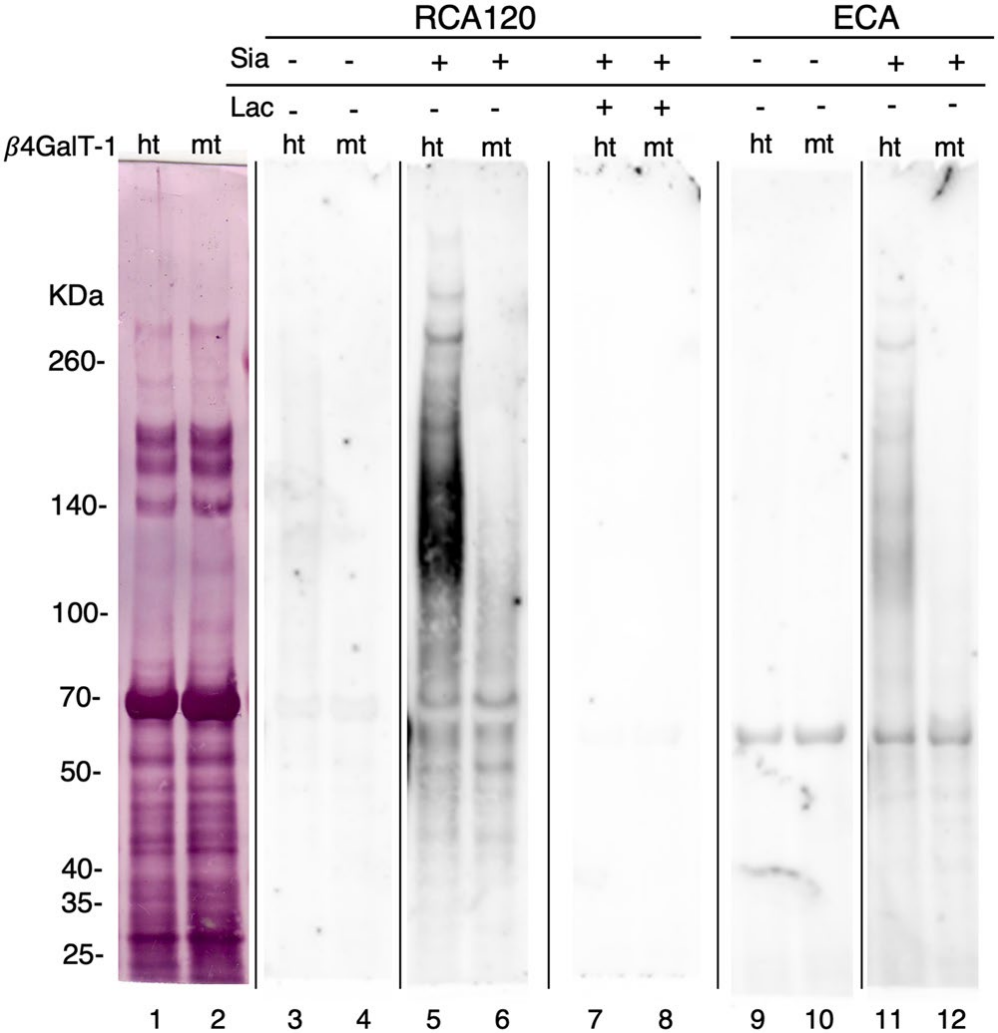
Lectin blot of lineage-negative bone marrow (BM) cells. Lanes 1, 2: lineage-negative BM cells (2.6 × 10^4^ cells) stained with Colloidal Gold. Lanes 3–12: lineage-negative BM cells (2.6 × 10^4^ cells) reacted with RCA-120 (lanes 3–8) or ECA (lanes 9–12). Lanes 5–6 and 11–12 were digested with sialidase, while lanes 7–8 were digested with sialidase and blocked with lactose. Lanes 1, 3, 5, 7, 9, 11 are *β4GalT-1*^+/−^ cells (ht), while lanes 2, 4, 6, 8, 10, 12 are *β4GalT-1*^−/−^ cells (mt). Lanes 1–12 were from the same gel. A representative lectin blot of three mice per genotype is shown.

To examine the galactosyl carbohydrate residues in HSPCs, lineage-negative BM cells were analyzed by a lectin blot using RCA120 and ECA, which specifically recognize Galβ1-4GlcNAc (Fig. 6). Since most proteins are sialylated at the non-reducing carbohydrate terminus, RCA120- and ECA-reactive bands were rarely detected in both *β4GalT-1*^+/−^ and *β4GalT-1*^−/−^ lineage-negative BM cells. However, digestion of cells with sialidase generated strong and smeary RCA120- and ECA-reactive bands between 100 and 200 kDa in *β4GalT-1*^+/−^ lineage-negative cells, but not in *β4GalT-1*^−/−^ lineage-negative cells (Fig. 6). This binding specificity was confirmed by the addition of lactose to specifically block RCA120 binding to Galβ1-4GlcNAc. Considering that the galactosyl residues in high-molecular-weight glycoproteins were lost in lineage-negative BM cells from *β4GalT-1*^−/−^ mice, these results suggested that they significantly promoted homing and engraftment.

### Colony formation by transplanted *Gne* (V572L) BM cells

**Figure 7:**
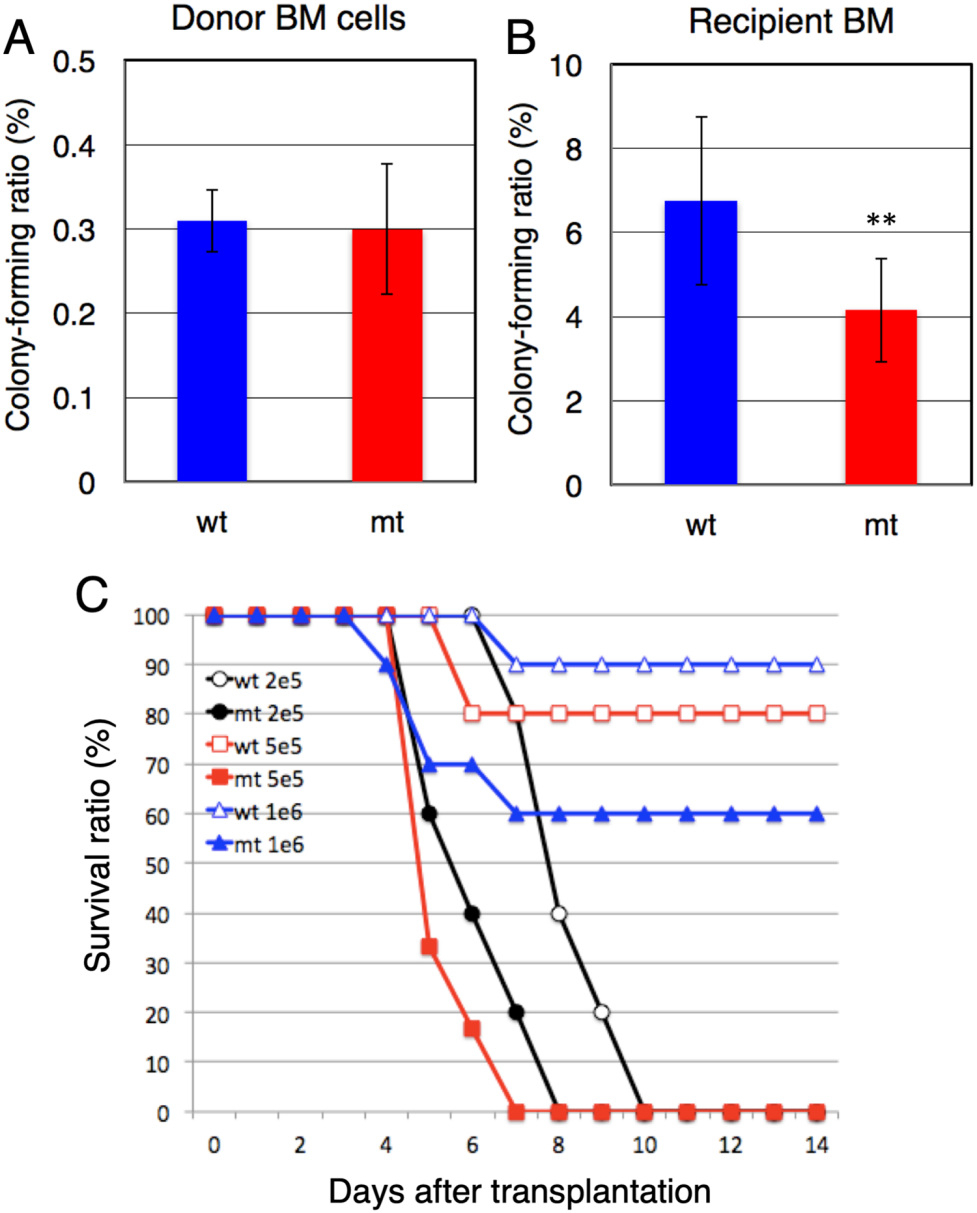
Homing of transplanted *Gne* (V572L) bone marrow (BM) cells and survival of recipient mice. (**A**) Colony-forming ratios of BM cells obtained from wild-type (wt, n = 4) and *Gne* (V572L) mice (mt, n = 4) prior to transplantation. (**B**) Colony-forming ratios of BM cells obtained from wild type recipient mice transplanted with wild-type (wt, n = 12) and *Gne* (V572L) BM cells (mt, n = 11), as measured 24 h after transplantation. Blue bars, wild-type cells; red bars, *Gne* (V572L) cells. Error bars indicate the S.D. **p < 0.01. (**C**) Survival of wild-type recipient mice transplanted with 2  × 10^5^ (black lines; wt, n = 5, mt, n = 5), 5 × 10^5^ (red lines; wt, n = 5, mt, n = 6), and 1 × 10^6^ (blue lines; wt, n = 10, mt, n = 10) *Gne* (V572L) (closed symbols) and wild-type cells (open symbols).

Sialic acids are well known to modify non-reducing terminal carbohydrates, including the galactosyl residues in glycoproteins and glycolipids. Notably, the lack of GNE, a key enzyme in sialic acid biosynthesis, is an embryonic lethal mutation^37^. However, mice with a V572L point mutation in GNE survive for several months, but suffer from a nephrotic-like syndrome because of severe hyposialylation of podocyte glycoproteins^38^. Colony formation by BM cells obtained from such mice was comparable with that of wild-type cells, suggesting that BM cell proliferation and differentiation were normal (Fig. 7A). However, colony-forming transplanted *Gne* (V572L) cells were about 60% as abundant in recipient BM cells as transplanted wild type cells (Fig. 7B).

The survival of lethally irradiated wild type mice clearly depended on the number of *Gne* (V572L) BM cells transplanted (Fig. 7C). For example, survival was lower in mice transplanted with 1  × 10^6^ *Gne* (V572L) BM cells (60%) than that in mice transplanted with the same number of wild-type cells (90%). The difference in survival was even larger (0% vs 80%) when 5 × 10^5^ cells were transplanted. However, all mice died within 10 days when 2 × 10^5^ cells were transplanted. Collectively, these data indicated that homing was mildly impaired in *Gne* (V572L) BM cells, suggesting that sialyl residues ligated to Galβ1-4GlcNAc also played a role in homing and engraftment, which was diminished by the *Gne* (V572L) point mutation.

## Discussion

Colony-forming activity of HSPCs and the number of pHSC (short-term [ST]-HSC) were not different between *β4GalT-1*^−/−^ BM cells and *β4GalT-1*^+/−^ BM cells. Although the number of functional HSC can be quantitatively examined using *in vivo* limiting dilution assay^39^ by BMT of serial diluted BM cells, it was difficult to estimate its number in *β4GalT-1*^−/−^ BM cells because homing and engraftment to the BM was severely impaired. In the differentiation pathway of HSC, the number of MPP was higher and those of MEP and CLP were lower in *β4GalT-1*^−/−^ BM cells than in *β4GalT-1*^+/−^ BM cells. These results suggest that HSC differentiation was disturbed to some extent downstream of MPP. Although the number of MPP increased in *β4GalT-1*^−/−^ BM cells, colony-forming activity of HSPCs was comparable between *β4GalT-1*^−/−^ and *β4GalT-1*^+/−^BM cells. Therefore, the disturbed differentiation downstream of MPP did not seem to have an influence on our findings that homing and engraftment of *β4GalT-1*^−/−^HSPCs after BMT were severely impaired. Colony-forming activity of HSPCs from *β4GalT-1*^−/−^ fetal liver cells slightly increased. These results indicated that the number, proliferation activity, or both of fetal liver HSPCs in *β4GalT-1*^−/−^ mice were slightly enhanced, not reduced, which also did not seem to affect the homing and engraftment of transferred *β4GalT-1*^−/−^fetal liver HSPCs.

Several cell adhesion systems such as VLA-4/VCAM-1 and CXCR4/SDF-1 play a major role in HSPC homing and engraftment to the BM, while other systems such as LFA-1/ICAM-1 and sLe^x^/endothelial selectins play a supportive role. However, these systems interact to ensure efficient homing and engraftment. As *β4GalT-1*^−/−^ mice also have compromised biosynthesis of selectin ligands and reduced inflammatory reactions^8^, the contribution of sLe^x^/endothelial selectins to HSPC homing and engraftment must also be impaired. However, lethally irradiated mice deficient in both P- and E-selectin survive when transplanted with at least 5  × 10^5^ BM cells^29^. Furthermore, homing in these mice deficient in both P- and E-selectin is reduced by approximately 50% compared with that in wild-type recipient mice^29^. These results strongly suggest that the defect in the sLe^x^/endothelial selectins system alone cannot explain the loss of homing and engraftment from *β4GalT-1*^−/−^ BM cells. Furthermore, the present results imply that a novel cell adhesion system based on galactosyl carbohydrates promotes HSPC homing and engraftment following transplantation. On the other hand, the abundance of colony-forming transplanted *Gne* (V572L) cells in recipient tissues, as well as the survival of lethally irradiated recipient mice transplanted with such cells, was highly similar to that previously observed in P/E-selectin double-knockout mice^29^. These results suggest that the impaired homing and engraftment of *Gne* (V572L) cells is due to the defect in the sLe^x^/endothelial selectins system. Alternatively, the mild phenotype of *Gne* (V572L) mice may be attributed to reduced, but not abolished, GNE activity.

During the latter half of mouse embryonic development until birth, hematopoiesis mainly occurs in the fetal liver^35,36^. Neonatal migration of HSPCs from the fetal liver to the adult BM seems to be normal in *β4GalT-1*^−/−^ mice, because colony-forming activity of HSPCs and ST-HSC populations in the adult BM were comparable between *β4GalT-1*^+/−^ and *β4GalT-1*^−/−^ mice. However, homing and engraftment of immature fetal liver HSPCs of *β4GalT-1*^−/−^mice to the adult recipient BM was impaired similar to that in the BM HSPCs of *β4GalT-1*^−/−^mice. These results suggest that fetal liver HSPCs and adult BM HSPCs use the similar galactosyl residues in homing and engraftment to the BM.

Lectin blots show that galactosyl residues in high-molecular-weight glycoproteins were lost in lineage-negative BM cells from *β4GalT-1*^−/−^ mice. Accordingly, these glycoproteins are good candidates as critical regulators of HSPC homing and engraftment. We noted that integrins such as integrin α4, α5, αL, and β1 are larger than 100 kDa and contain many possible glycosylation sites, some of which are actually *N*-glycosylated (Glycoprotein Database, http://jcggdb.jp/rcmg/gpdb/index.action). Therefore, it is possible that the function of VLA-4 (α4β1 integrin), VLA-5 (α5β1 integrin), or LFA-1 (αLβ2 integrin) in HSPC homing and engraftment is compromised by β4GalT-1 and GNE deficiency. Another possibility is that β4GalT-1 and GNE deficiency may disrupt the function of unknown carbohydrate ligands that regulate homing and engraftment. Thus, further studies are necessary to fully elucidate the role of carbohydrate residues in HSPC homing and engraftment.

In clinical applications of BMT, especially in cord blood transplantation, it is essential to enhance the efficiency of HSPC homing and engraftment. The present study suggests the possibility of modifying carbohydrate structures on the surface of HSPCs to improve their homing and engraftment to the BM in clinical application. Indeed, a recent study demonstrated that the *ex vivo* fucosylation of cord blood cells improved their homing abilities, leading to faster neutrophil and platelet engraftments^40^. Accordingly, it might be possible that enforced galactosylation and sialylation of HSPCs would also improve their homing and engraftment to the BM.

In conclusion, we have demonstrated that β4GalT-1 activity in donor BM cells, but not recipient mice, is critical for hematopoietic reconstitution and homing/engraftment to the BM after transplantation. However, BM cells from *Gne* (V572L) mice only showed relatively mild impairment. The deficiency of BM cells in sLe^x^/endothelial selectins system might explain the defect of BM cells from *Gne* (V572L) mice, but cannot explain the defect of *β4GalT-1*^−/−^ BM cells. Collectively, these data suggest that a novel cell adhesion system containing galactosyl or sialyl residues or both may promote homing and engrafting of HSPCs to the BM.

## Materials and Methods

### Mice

*β4GalT-1*^−/−^ mice on a mixed 129/Sv and C57BL/6 genetic background, and mice with a V572L point mutation in GNE [*Gne* (V572L) mice] on a C57BL/6 background were described previously^34,38^. To produce GFP-labeled BM cells, these mice were crossed with *CAGGFP* mice on a C57BL/6 background, which were kindly provided by Dr. Okabe at Osaka University^41^. NOD/SCID mice and pseudo-pregnant ICR mice were purchased from Charles River Japan and CLEA Japan, Inc., respectively. Animal experiments were conducted in accordance with the Fundamental Guidelines for Proper Conduct of Animal Experiment and Related Activities in Academic Research Institutions under the jurisdiction of the Ministry of Education, Culture, Sports, Science and Technology of Japan, and were approved by the Committee on Animal Experimentation at Kanazawa University and Kyoto University, Japan.

### Preparation of BM cells

BM cells were harvested by aseptically flushing the femur and tibia using a 22-gauge needle with Dulbecco’s modified Eagle’s medium (DMEM, Life Technologies, Grand Island, NY, USA) containing 5% fetal calf serum (FCS). The obtained cell suspension was filtered through a 70-μm mesh, treated with 140 mM NH_4_Cl in 17 mM Tris-HCl (pH 7.2) buffer for 5 min to lyse red blood cells, washed, and suspended in DMEM with 5% FCS for BMT and the colony formation assay.

### Preparation of fetal liver cells

Oocytes from *β4GalT-1*^+/−^ mice with the *CAGGFP* gene in homozygotes were fertilized *in vitro* by sperms from *β4GalT-1*^−/−^ mice and fertilized two-cell stage eggs were transferred to the oviduct of pseudo-pregnant ICR females. Embryos were collected at E14.5 and fetal livers were prepared by crushing using a plunger on 70-μm strainer in Ca^2+^- and Mg^2+^-free phosphate-buffered saline (PBS) containing 3% FCS. Fetal liver cells were collected after centrifugation, suspended in Hanks’ solution, and passed through a 40-μm strainer for transplantation and colony formation assay^42^.

### Flow cytometry

Flow cytometry was performed using a FACS Aria II and Aria IIIu cell sorter (BD Biosciences, Franklin Lakes, NJ, USA) and analyzed using FlowJo software (Tree Star). BM cells were collected from the bilateral femur and tibia by flushing using a 22-gauge needle in PBS containing 3% FCS. Cells were passed through a 70-μm strainer and treated with 140 mM NH_4_Cl, 17 mM Tris-HCl (pH 7.2) buffer for 5 min to lyse red blood cells. After washing in RPMI-1640 (Life Technologies) three times, the cells were passed through a 40-μm strainer to exclude cell aggregates before analysis. For BM cell analysis, the cells were stained with combinations of antibodies against the following surface markers: c-Kit (clone 2B8; Thermo Fisher Scientific, Waltham, MA, USA), Sca-1 (clone D7, BD Biosciences), Flk2 (clone A2F10, Thermo Fisher Scientific), CD150 (clone TC15-12F12.2, BioLegend, San Diego, CA, USA), CD34 (clone RAM34, Thermo Fisher Scientific), and Fc blocker CD16/32 (clone 93, Thermo Fisher Scientific). The lineage markers used for Fig. 1 included Ter-119 (clone TER-119, BioLegend), B220 (clone RA3-6B2, BioLegend), CD3e (clone 145-2C11, BioLegend), CD4 (clone GK1.5; BioLegend), CD8a (clone 53–6.7; BioLegend), Gr-1 (clone RB6-8C5, BioLegend), CD11b (clone M1/70, BD Biosciences), and IL-7Rα (clone A7R34, Thermo Fisher Scientific). The lineage markers (BioLegend 133313) used for Fig. 2 included CD3 (clone 17A2), Gr-1 (clone RB6-8C5), CD11b (clone M1/70), B220 (clone RA3-6B2), TER-119 (clone Ter-119). Antibody staining was performed at 4 °C and the cells were incubated for 30 min, except for cells stained with CD34, which were incubated for 90 min. Before analysis, the cells were stained with SYTOX Red Dead Cell Stain (Life Technologies) to assess viability per the manufacturer’s recommendations.

### BM transplantation

Recipient mice were lethally irradiated with 9.5 Gy (Hitachi MBR-1520R), and transplanted 3–6 h after with GFP-labeled BM cells in Hanks’ solution using IV-BMT or IBM-BMT^33^. To assess survival and homing, 2  × 10^7^ and 2–5 × 10^6^ cells were transplanted, respectively. GFP-labeled fetal liver cells (5  × 10^6^ to 1  × 10^7^cells) were also transplanted through the orbital vein into lethally irradiated wild-type recipient mice. NOD/SCID mice were similarly treated except that a 3.5-Gy dose was used, as these mice are very sensitive to radiation.

### Colony formation assay

Specimens of the femur and spleen were prepared from recipient mice 3 h and 24 h after transplantation. BM cells and splenocytes collected from these specimens were filtered through a 70-μm mesh, suspended in RPMI-1640 medium, and mixed with MethoCult^TM^ GF-M3434 medium (STEMCELL Technologies, Vancouver, Canada) according to the manufacturer’s procedure. After incubation at 37 °C and 5% CO_2_ for 7–11 days, GFP-positive donor-derived colonies were counted. GFP-positive colonies obtained from recipient tissues were normalized to the total BM cells in the recipient, considering that BM cells in the femur specimen constitute approximately 6.7% of all BM cells in a mouse^43^. The colony-forming ratio of the BM was calculated as the number of GFP-positive colonies per recipient mouse relative to the number of transplanted donor cells (2–5 × 10^6^). Similarly, the colony-forming ratio in the spleen was calculated as the number of GFP-positive colonies per total spleen relative to the number of transplanted donor cells. Colonies formed by untransplanted donor BM cells were also quantified. When fetal liver cells were transplanted, GFP-positive colonies obtained from recipient femurs were normalized to 3 × 10^7^ total BM cells in the femur of recipient mice. Colonies formed by untransplanted donor fetal liver cells were also quantified.

### Histological analysis

Femurs of wild-type recipient mice 24 h after transplantation of GFP-labeled* β4GalT-1*^+/−^and* β4GalT-1*^−/−^ BM cells were prepared, fixed in 4% paraformaldehyde for 5 h, and equilibrated in 30% sucrose/PBS. Fixed bone samples were embedded in SCEM-L1 (SECTION-LAB, Hiroshima, Japan) and frozen in cooled hexane with liquid nitrogen gas (N_2_). Cryostat sections (8 mm thick) were generated using Kawamoto’s film method^44^. The sections were stained with 4′,6-diamidino-2-phenylindole (DAPI) and observed using a BZ-X fluorescence microscope (Keyence, Osaka, Japan).

### Preparation of lineage-negative cells

BM cells (1 × 10^7^ cells/mL) were prepared in PBS containing 3% FCS, incubated on ice for 15 min with 2.5 μg anti-mouse CD16/CD32 (Fc Block, BD Biosciences) per 10^7^ cells, and then incubated on ice for 15 min with Biotin Mouse Lineage Depletion Cocktail (BD Biosciences) consisting of biotinylated monoclonal anti-mouse CD3e, CD11b, CD45R/B220, Ly-6G, Ly-6C, and TER-119. Subsequently, the mixture was washed with 10 volumes of PBS containing 0.5% bovine serum albumin and 2 mM ethylenediaminetetraacetic acid, and centrifuged. The resulting cell pellets were mixed with Streptavidin Particles Plus-DM (BD Biosciences), incubated at 6–12 °C for 30 min, and lineage-positive cells were removed using BD IMagnet (BD Biosciences) according to the manufacturer’s protocol. The depleted fractions were then used as lineage-negative BM cells, which have about 10-fold higher colony-forming activity than crude BM cells.

### Lectin blot

Lineage-negative BM cells (2.6 × 10^4^ cells) were suspended in NuPAGE LDS Sample Buffer (Thermo Fisher Scientific), electrophoresed on SuperSep Ace 5–12% precast gels (Wako Pure Chemical Industries, Tokyo, Japan), and transferred to polyvinylidene difluoride membranes by electroblotting (Bio-Rad, Hercules, CA, USA). Lectin blotting was performed as described previously^45^. In brief, the membranes were washed in blocking buffer (10 mM Tris-HCl pH 7.4, 0.15 M NaCl, and 0.05% Tween 20), and immersed for 1 h with gentle shaking in blocking buffer containing 2 μg/mL biotinylated RCA120 (Seikagaku Corporation, Tokyo, Japan) or 5 μg/mL biotinylated ECA (Seikagaku Corporation). The membranes were then washed again, immersed for 30 min with gentle shaking in blocking buffer containing 1:2000 avidin conjugated to horseradish peroxidase, washed, incubated with ImmunoStar (Wako Pure Chemical Industries), and visualized with ChemiStage (Kurabo, Osaka, Japan). Total proteins were also stained with Colloidal Gold for comparison (Bio-Rad). In the RCA120 blots, certain membranes were treated with 0.05 U/mL sialidase (Roche, Mannheim, Germany) at 37 °C for 30 min before probing with lectins, or with 0.2 M lactose during probing with lectins.

### Statistical analysis

Differences with two-sided *p* < 0.05 were deemed statistically significant, as evaluated using the Student’s *t*-test. Results are reported as mean ± standard deviation (S.D.).

## Supplementary information

Supplementary Information

## References

[1] Lowe JB (2003). Glycan-dependent leukocyte adhesion and recruitment in inflammation. Curr. Opin. Cell Biol..

[2] McEver RP, Moore KL, Cummings RD (1995). Leukocyte trafficking mediated by selectin-carbohydrate interactions. J. Biol. Chem..

[3] Frenette PS, Mayadas TN, Rayburn H, Hynes RO, Wagner DD (1996). Susceptibility to infection and altered hematopoiesis in mice deficient in both P- and E-selectins. Cell.

[4] Bullard DC (1996). Infectious susceptibility and severe deficiency of leukocyte rolling and recruitment in E-selectin and P-selectin double mutant mice. J. Exp. Med..

[5] Mitsuoka C (1998). Identification of a major carbohydrate capping group of the L-selectin ligand on high endothelial venules in human lymph nodes as 6-sulfo sialyl Lewis X. J. Biol. Chem..

[6] Arbonés ML (1994). Lymphocyte homing and leukocyte rolling and migration are impaired in L-selectin-deficient mice. Immunity.

[7] Homeister JW (2001). The alpha(1,3)fucosyltransferases FucT-IV and FucT-VII exert collaborative control over selectin-dependent leukocyte recruitment and lymphocyte homing. Immunity.

[8] Asano M (2003). Impaired selectin-ligand biosynthesis and reduced inflammatory responses in beta-1,4-galactosyltransferase-I-deficient mice. Blood.

[9] Mori R, Kondo T, Nishie T, Ohshima T, Asano M (2004). Impairment of skin wound healing in beta-1,4-galactosyltransferase-deficient mice with reduced leukocyte recruitment. Am. J. Pathol..

[10] Aizawa S, Tavassoli M (1987). *In vitro* homing of hemopoietic stem cells is mediated by a recognition system with galactosyl and mannosyl specificities. Proc. Natl. Acad. Sci. USA.

[11] Lapidot T, Dar A, Kollet O (2005). How do stem cells find their way home?. Blood.

[12] Wilson A, Trumpp A (2006). Bone-marrow haematopoietic-stem-cell niches. Nat. Rev. Immunol..

[13] Heazlewood SY, Oteiza A, Cao H, Nilsson SK (2014). Analyzing hematopoietic stem cell homing, lodgment, and engraftment to better understand the bone marrow niche. Ann. N. Y. Acad. Sci..

[14] Vermeulen M (1998). Role of adhesion molecules in the homing and mobilization of murine hematopoietic stem and progenitor cells. Blood.

[15] van der Loo JC (1998). VLA-5 is expressed by mouse and human long-term repopulating hematopoietic cells and mediates adhesion to extracellular matrix protein fibronectin. J. Clin. Invest..

[16] Papayannopoulou T, Craddock C, Nakamoto B, Priestley GV, Wolf NS (1995). The VLA4/VCAM-1 adhesion pathway defines contrasting mechanisms of lodgement of transplanted murine hemopoietic progenitors between bone marrow and spleen. Proc. Natl. Acad. Sci. USA.

[17] Peled A (2000). The chemokine SDF-1 activates the integrins LFA-1, VLA-4, and VLA-5 on immature human CD34(+) cells: role in transendothelial/stromal migration and engraftment of NOD/SCID mice. Blood.

[18] Potocnik AJ, Brakebusch C, Fässler R (2000). Fetal and adult hematopoietic stem cells require beta1 integrin function for colonizing fetal liver, spleen, and bone marrow. Immunity.

[19] Katayama Y, Hidalgo A, Peired A, Frenette PS (2004). Integrin alpha4beta7 and its counterreceptor MAdCAM-1 contribute to hematopoietic progenitor recruitment into bone marrow following transplantation. Blood.

[20] Avigdor A (2004). CD44 and hyaluronic acid cooperate with SDF-1 in the trafficking of human CD34+ stem/progenitor cells to bone marrow. Blood.

[21] Gu Y (2003). Hematopoietic cell regulation by Rac1 and Rac2 guanosine triphosphatases. Science.

[22] Grassinger J (2009). Thrombin-cleaved osteopontin regulates hemopoietic stem and progenitor cell functions through interactions with alpha9beta1 and alpha4beta1 integrins. Blood.

[23] Kimura T (2004). The sphingosine 1-phosphate receptor agonist FTY720 supports CXCR4-dependent migration and bone marrow homing of human CD34+ progenitor cells. Blood.

[24] Hoggatt J, Singh P, Sampath J, Pelus LM (2009). Prostaglandin E2 enhances hematopoietic stem cell homing, survival, and proliferation. Blood.

[25] Driessen RL, Johnston HM, Nilsson SK (2003). Membrane-bound stem cell factor is a key regulator in the initial lodgment of stem cells within the endosteal marrow region. Exp. Hematol..

[26] Peled A (1999). Dependence of human stem cell engraftment and repopulation of NOD/SCID mice on CXCR4. Science.

[27] Kawabata K (1999). A cell-autonomous requirement for CXCR4 in long-term lymphoid and myeloid reconstitution. Proc. Natl. Acad. Sci. USA.

[28] Ara T (2003). Long-term hematopoietic stem cells require stromal cell-derived factor-1 for colonizing bone marrow during ontogeny. Immunity.

[29] Frenette PS (1998). Endothelial selectins and vascular cell adhesion molecule-1 promote hematopoietic progenitor homing to bone marrow. Proc. Natl. Acad. Sci. USA.

[30] Katayama Y (2003). PSGL-1 participates in E-selectin-mediated progenitor homing to bone marrow: evidence for cooperation between E-selectin ligands and alpha4 integrin. Blood.

[31] Chen JY (2016). Hoxb5 marks long-term haematopoietic stem cells and reveals a homogenous perivascular niche. Nature.

[32] Akashi K, Traver D, Miyamoto T, Weissman IL (2000). A clonogenic common myeloid progenitor that gives rise to all myeloid lineages. Nature.

[33] Kushida T (2001). Intra–bone marrow injection of allogeneic bone marrow cells: a powerful new strategy for treatment of intractable autoimmune diseases in MRL/lpr mice. Blood.

[34] Asano M (1997). Growth retardation and early death of beta-1,4-galactosyltransferase knockout mice with augmented proliferation and abnormal differentiation of epithelial cells. EMBO J..

[35] Inlay MA (2014). Identification of multipotent progenitors that emerge prior to hematopoietic stem cells in embryonic development. Stem Cell Reports.

[36] Gao X, Xu C, Asada N, Frenette PS (2018). The hematopoietic stem cell niche: from embryo to adult. Development.

[37] Schwarzkopf M (2002). Sialylation is essential for early development in mice. Proc. Natl. Acad. Sci. USA.

[38] Ito M (2012). Glycoprotein hyposialylation gives rise to a nephrotic-like syndrome that is prevented by sialic acid administration in GNE V572L point-mutant mice. PLoS ONE.

[39] Purton LE, Scadden DT (2007). Limiting factors in murine hematopoietic stem cell assays. Cell Stem Cell.

[40] Popat U (2015). Enforced fucosylation of cord blood hematopoietic cells accelerates neutrophil and platelet engraftment after transplantation. Blood.

[41] Okabe M, Ikawa M, Kominami K, Nakanishi T, Nishimune Y (1997). ‘Green mice’ as a source of ubiquitous green cells. FEBS Letters.

[42] Gudmundsson KO, Stull SW, Keller JR (2012). Transplantation of mouse fetal liver cells for analyzing the function of hematopoietic stem and progenitor cells. Methods Mol. Biol..

[43] Boggs DR (1984). The total marrow mass of the mouse: A simplified method of measurement. American Journal of Hematology.

[44] Kawamoto T (2003). Use of a new adhesive film for the preparation of multi-purpose fresh-frozen sections from hard tissues, whole-animals, insects and plants. Arch. Histol. Cytol..

[45] Nishie T (2010). Beta4-galactosyltransferase-5 is a lactosylceramide synthase essential for mouse extra-embryonic development. Glycobiology.

