# Supplementary material for: Galactosyl carbohydrate residues on hematopoietic stem/progenitor cells are essential for homing and engraftment to the bone marrow
Source: Sci Rep. 2019 May 9;9:7133. doi: 10.1038/s41598-019-43551-6 (PMC6509332; doi:10.1038/s41598-019-43551-6)
Supplement: Supplementary file 1 — Supplementary Information [file 41598_2019_43551_MOESM1_ESM.pdf]

Supplementary Information

Galactosyl carbohydrate residues on hematopoietic stem/progenitor cells are essential for homing and engraftment to the bone marrow

Soichiro Takagaki,[1] Rieko Yamashita,[2,3] Noriyoshi Hashimoto,[1] Kazushi Sugihara,[1,2] Kanako Kanari,[2] Keisuke Tabata,[1] Toshikazu Nishie,[1] Shogo Oka,[3] Masanori, Miyanishi,[4] Chie Naruse, [1,2] and Masahide Asano[1,2,*]

[1]Division of Transgenic Animal Science, Advanced Science Research Center, Kanazawa University, Kanazawa 920-8640, Japan

[2]Institute of Laboratory Animals, Graduate School of Medicine, Kyoto University, Kyoto 606-8501, Japan

[3]Department of Biological Chemistry, Human Health Sciences, Graduate School of Medicine, Kyoto University, Kyoto 606-8507, Japan

[4]Laboratory for Organismal Patterning, RIKEN Center for Biosystems Dynamics Research, Kobe 650-0047, Japan

***Correspondence:** Masahide Asano, Ph.D., E-mail: asano@anim.med.kyoto-u.ac.jp

Supplemental figure legends

Figure S1. Gating scheme for hematopoietic stem/progenitor cells (HSPCs)

Representative flow cytometry gating to isolate MPP, CMP, CLP, GMP, and MEP from *β4GalT-1*$^{+/-}$ (ht) and *β4GalT-1*$^{-/-}$ (mt) BM cells.

Fig S1

ht

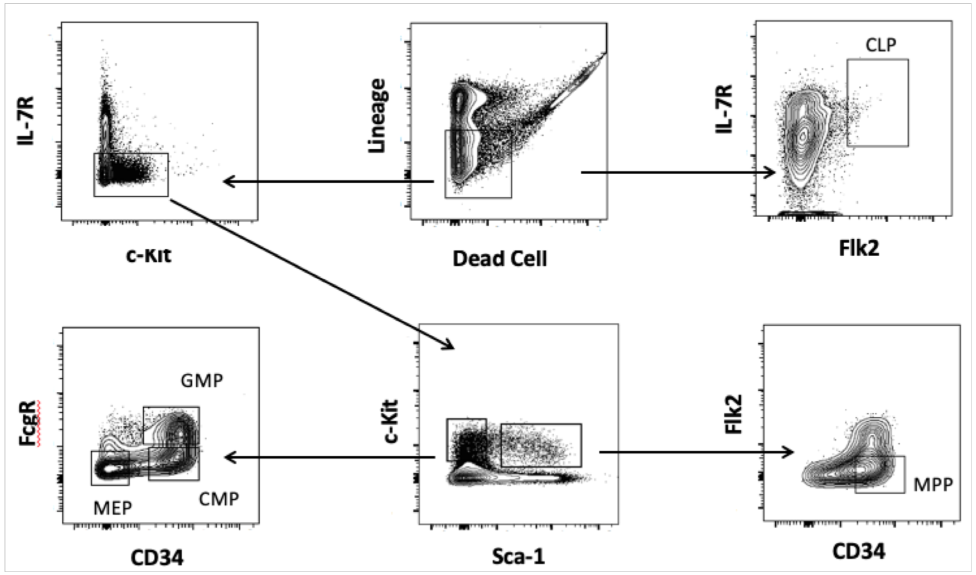
IL-7R
c-Kit
Lineage
Dead Cell
IL-7R
CLP
Flk2
FcgR
GMP
MEP
CMP
CD34
c-Kit
Sca-1
Flk2
MPP
CD34

mt

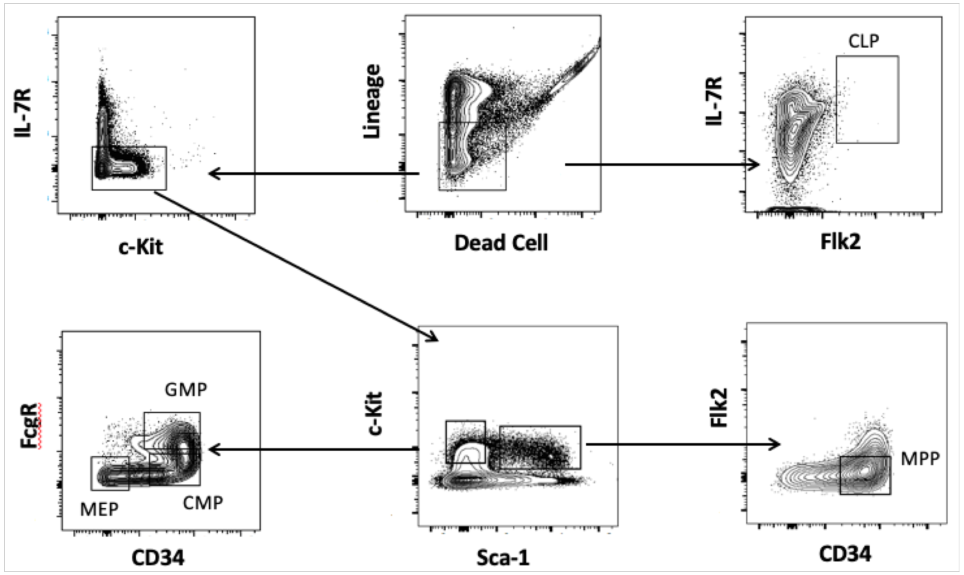
IL-7R
c-Kit
Lineage
Dead Cell
IL-7R
CLP
Flk2
FcgR
GMP
MEP
CMP
CD34
c-Kit
Sca-1
Flk2
MPP
CD34
